# Epidemiology and prevention of drowning in Iran

**Published:** 2019-07

**Authors:** Shademan Reza Masouleh, Arezoo Monfared

**Affiliations:** ^*a*^Department of Nursing, Guilan University of Medical Sciences, Rasht, Iran.; ^*b*^Guilan Road Trauma Research Center, Guilan University of Medical Sciences, Rasht, Iran.; ^*c*^Department of Nursing, Islamic Azad University, Rasht Branch, Guilan, Iran.

## Abstract

**Background:**

Drowning as a major health problem is one of the most important causes of mortality. The purpose of this study is to review the epidemiology and prevention of drowning in Iran.

**Methods:**

The present study was conducted with reviewing of the prior researches, WHO reports, scientific web sites, and studies on drowning epidemiology occurring in some countries, especially Iran, in recent years. Accordingly, the databases including Google Scholar, Magiran, and SID were searched with keywords of drowning, epidemiology, prevention, drowning prevention, and related articles since 2001 were included in our study.

**Results:**

Of 1026 people drowned in Iran in 2017, 168 were female and 858 male. Coastal waters of Guilan accounted for 88% of the drowning cases at sea during 2012-16.The age group of 20-24 years had the highest number of drowning cases, followed by the age group of 15-19 years, and 25-29 years. The highest number of drowning happened in 23 July-22 August (34.6%), 22 June -22 July (22.3%), and 23 August-22 September (19.8%). A study conducted in the northern provinces of Iran showed the death rate due to drowning was estimated 2.9 per 100,000 populations, and the number of years of life lost was 4110 years.

**Conclusions:**

As the highest number of drowning occurs in seas and summer, and the highest number of years of life lost happens in the age group of adolescents and young people, it seems that these features need paying more attention in planning and designing the necessary interventions. Considering the main risk factors for drowning, integrated effort and management developing and regulating the rules and standards of the swimming zones and, most importantly, developing education and skills and managing drowning and training the resuscitation skills to reduce the resulting complications are effective in implementing safe interventions and declining drowning-related deaths.

**Keywords:**

Drowning, Epidemiology, Prevention, Iran, Accident

